# Immune-mediated polyarthritis in dogs: Are corticosteroids the best bet?

**DOI:** 10.18849/ve.v8i1.584

**Published:** 2023-01-25

**Authors:** Hannah Walker+

**Keywords:** IMMUNE-MEDIATED POLYARTHRITIS, IMPA, TYPE I, TYPE 1, IDIOPATHIC, POLYARTHROPY, CANINE, CANIS FAMILIARIS, CORTICOSTEROIDS, IMMUNOSUPPRESSANTS, IMMUNOSUPPRESSIVE, IMMUNOMODULATORS, TREATMENT, MEDICATION, IMMUNE-MEDIATED, PREDNISONE, PREDNISOLONE

## Abstract

**PICO question:**

In dogs with type I immune-mediated polyarthritis (IMPA), is sole treatment with other immunosuppressive agents as effective as treatment with corticosteroids at reducing clinical signs?

**Clinical bottom line:**

**Category of research:**

Treatment.

**Number and type of study designs reviewed:**

One pragmatic open-label randomised controlled clinical trial.

**Strength of evidence:**

Weak.

**Outcomes reported:**

In the single randomised controlled clinical trial reviewed, 7/10 (70%) of dogs in both treatment groups (prednisone or cyclosporine), were reported to have shown resolution of owner-reported symptoms, clinical symptoms and improved locomotor scores and cytologic signs of disease at the end of the 90 day trial period. Of the remaining dogs, 2/3 cyclosporine treated dogs required change to prednisone, and 2/3 prednisone treated dogs required combination therapy to achieve clinical response.

**Conclusion:**

There is insufficient evidence to support the use of alternative immunosuppressive agents in place of corticosteroids for the treatment of IMPA type I. Further controlled clinical trials are needed before a change to clinical practice can be considered.

## How to apply this evidence in practice

The application of evidence into practice should take into account multiple factors, not limited to: individual clinical expertise, patient’s circumstances and owners’ values, country, location or clinic where you work, the individual case in front of you, the availability of therapies and resources.

Knowledge Summaries are a resource to help reinforce or inform decision making. They do not override the responsibility or judgement of the practitioner to do what is best for the animal in their care.

## Clinical scenario

A dog presents to the clinic with severe bilateral front limb lameness, widespread joint pain and inflammation, pyrexia, inappetence and lethargy. Clinical indications suggest the dog is likely experiencing idiopathic immune-mediated polyarthritis (IMPA type I). This speculation is confirmed through blood tests, x-rays, arthrocentesis, and cytological results. The patient has a history of iatrogenic Cushing’s syndrome as a result of previous corticosteroid treatment. To avoid a recurrence of iatrogenic Cushing’s, you wonder if any alternative immunosuppressive agents could provide as effective treatment for IMPA type I as prednisone.

## The evidence

One relevant paper was found, describing a randomised controlled clinical trial comparing an alternative immunosuppressant to a corticosteroid for the treatment of IMPA type I. Rhoades et al. (2016) compared the use of prednisone and cyclosporine in a population of 20 dogs with IMPA type I presenting to a Californian veterinary hospital. The study had restricted selection criteria, pathologist blinding, randomisation of treatment allocation and multiple objective and subjective outcome measures. Concurrent medication use was a potential confound. Although Rhoades et al. (2016) produced moderate evidence for an alternative immunosuppressant providing comparable treatment success to a corticosteroid, the power of this evidence was relatively weak and 2/10 (20%) of the cyclosporine group were ultimately switched to prednisone to achieve treatment success. Therefore, additional randomised controlled clinical trials with larger sample sizes are required to validate immunosuppressants as an alternative to corticosteroids in clinical practice.

## Summary of the evidence

**Rhoades et al. (2016):** 

Population	Dogs with type I (primary / idiopathic) immune-mediated polyarthritis. Diagnosis of primary (type I) immune-mediated polyarthritis with no evidence of secondary causes. 10 males (nine neutered and one entire) and 10 spayed females. 12 dogs over 15 kg and eight dogs under 15 kg. Five mixed breed dogs and 15 various pedigrees. Ages of dogs were not reported.
Sample size	20 client owned dogs.
Intervention details	Prednisone group n=10. Cyclosporine group n=10. Dogs were randomly allocated into two groups: Prednisone (starting at 1 mg/kg orally every 12 hours, tapered by 25% every 2–3 weeks). Cyclosporine (5 mg/kg orally every 12 hours). Allocation was determined by drawing cards from a hat: Simple (unrestricted) randomisation is usually only recommended for studies with large sample sizes due to risk of covariates leading to allocation bias. However, no significant difference in any of the variables measured was found between the two groups (P = 0.54–1.0) at the beginning of the study period, suggesting that this form of randomisation may have been sufficient. Clinical pathologists recording cytologic data were blinded to treatment group. Clinicians and owners could not be blinded due to the different administration, tapering and monitoring protocols for each medication. Authors attempted to reduce bias by ensuring only one clinician and owner provided reports for each dog.
Study design	Pragmatic open-label randomised controlled clinical trial.
Outcome studied	Data was collected at Day 0 (pre-treatment), 14, 45, and 90. Physical examination, survey data collection and cytologic analysis of multiple peripheral joint arthrocentesis samples were performed. An owner questionnaire provided data on: Adverse effects: polydipsia, polyuria, panting, polyphagia, vomiting and diarrhoea (4-point Likert scale). Perceived comfort, gait and degrees of physical activity, lameness and lethargy (5-point Likert scale). Perceived quality of life (5-point Likert scale). An overall mobility score was obtained by combining scores of owner-perceived comfort or gait and lameness. Clinician assessment provided data on: Variables including body weight, rectal temperature, hydration level, gait or lameness (5-point Likert scale). Signs of pain, grating, and swelling on 8 peripheral joints (carpi, tarsi, stifles, and elbows) (5-point Likert scale). An overall locomotion score was calculated by combining gait and joint effusion scores and signs of joint pain. Clinical pathologist assessment provided data on: Total and differential cell counts from synovial fluid smears obtained through multiple arthrocentesis, including the carpi and tarsi, scored using a standard scoring system described by Berg et al. (2009). In cases of IMPA, the carpus, stifle, and hock are the most commonly implicated joints and it is recommended that the carpi and tarsi are sampled as a minimum (Jacques et al., 2002; and Stull et al., 2008). Bilateral paired joint sampling is suggested to increase diagnostic accuracy (Stull et al., 2008). Mean inflammation score and mean neutrophilic inflammation score across all joints (5-point Likert scale). Maximum neutrophilic inflammation score, obtained from the joint in the set with the highest neutrophilic inflammation (5-point Likert scale). Treatment was recorded as a failure if the dog had to be changed to a different medication due to either: Lack of clinical improvement by day 14. Lack of cytologic improvement by day 45. Intolerable adverse effects.
Main findings (relevant to PICO question)	7/10 (70%) of dogs in the prednisone-treated group were reported to have achieved successful treatment. Of the three prednisone-treated dogs that did not achieve treatment success: Two dogs showed lack of clinical improvement by day 14. One dog was switched to a combination therapy (prednisone and cyclosporine), though never achieved cytologic improvement. The other dog died (cause unknown). One dog showed lack of cytologic improvement by day 45 and was successfully treated with a combination therapy (prednisone and azathioprine). By day 90, adverse effects was reported for nine dogs on prednisone treatment, including the two switched to combination therapy, but outcome data were only collected for the seven dogs on prednisone alone. 7/10 (70%) of dogs in the cyclosporine-treated group were also reported to have achieved successful treatment. Of the three cyclosporine-treated dogs that did not achieve treatment success: One dog developed diarrhoea by day 5 of treatment and was switched to prednisone. Prednisone treatment was successful. One dog showed lack of clinical improvement by day 14 and was switched to prednisone. Prednisone treatment was successful. One dog showed lack of cytologic improvement by day 45. Owner declined alternative immunosuppressant treatment, as clinical symptoms had been successfully resolved. Cyclosporine was consequently tapered, but it is unclear whether this tapering occurred during or after the trial period. One dog developed an infection on day 75 and cyclosporine treatment was discontinued, having achieved an acceptable clinical response. This dog was still considered a treatment success. For both treatment groups, 7 of 10 dogs were reported to have shown a significant decrease from baseline in mean joint neutrophilic inflammation score by day 90. Authors reported no significant difference in the change from baseline in mean inflammation score between the two treatment groups (p-value not stated). Authors reported no significant difference in mean and maximum joint neutrophilic inflammation scores on day 14 (P = ≥ 0.81), day 45 (P = ≥ 0.49) and day 90 (P = ≥ 0.85). Statistical significance was accepted when P = < 0.05. The authors used the Wilcoxon-Mann-Whitney test to compare distributions of clinical, owner-perceived and cytological scores relative to baseline between groups at different time points. Although the authors combined non-parametric tests with parametric by calculating standard deviation, non-parametric tests are often recommended for the analysis of scores obtained in medical trials due to their higher efficiency.
Limitations	Very small sample size limits power of statistical results and validity of conclusions. The authors did not calculate minimum sample sizes required to achieve sufficient power. Randomisation was conducted through the drawing of cards from a hat. This is a simple randomisation technique that does not account for confounding variables. The use of concurrent analgesics in the cyclosporine treatment group but not the prednisone group may have affected owner-reported and clinical results in the cyclosporine group. Additionally, other medications such as antacids and antiemetics were not controlled or reported and this may have affected measures of adverse effects in both groups. Researchers evaluating cytological improvement were blinded to the treatment, but clinicians and owners could not be blinded. This may have introduced data bias from clinician-reported and owner-reported signs and side-effects. There were limited correlations between owner-perceived mobility, clinician-assessed locomotion, and cytologic scores. This suggests that clinicians, owners, and pathologists were perceiving and reporting each dog’s improvement differently. A concurrent reliability study would clarify this lack of agreement. Joint radiography was not performed on all patients. Although radiographs are not always considered mandatory in the diagnosis of IMPA, radiographs allow for the exclusion of differential diagnoses (such as erosive immune-mediated polyarthritis, rheumatoid arthritis, polyarthritis-polymyositis syndrome and systemic lupus erythematosus) (Bennett, 1987). Therefore, it is plausible that differential conditions were actually the primary diagnosis for some individuals. Standard deviation bars in the data concerning owner-perceived mobility do not overlap by day 90, suggesting a significant difference in owner-scored mobility between prednisone and cyclosporine-treated dogs at the end of the study period, with cyclosporine dogs showing potentially significantly worse mobility scores. However, this difference was not reported in the text. The authors omitted some information from the figures, including numbers of dogs from which the data was obtained. When numbers of dogs are reported graphically, this does not always correlate with information provided with the text. These gaps in data are not explained. The authors did not obtain longitudinal data, so were not able to assess rates of relapse between the two groups. As relapse is common in immune-mediated polyarthritis (Bennett, 1987; Clements et al., 2004) information on relapse rates would have been helpful. The study design does not conform to CONSORT 2010 guidelines for an RCT (Schulz et al., 2010). The lack of methodological rigour in the study limits the power of conclusions drawn.

## Appraisal, application and reflection

Canine immune-mediated polyarthritis (IMPA) is a little understood condition (Stull et al., 2008). Symptoms include significant pain, lethargy, lameness, localised joint swelling, and pyrexia (Itoh et al., 2010; Jacques et al., 2002; and Johnson & Mackin, 2012). Currently, corticosteroids are the primary treatment for type I IMPA (Kohn, 2007; Innes, 2012; and Itoh et al., 2010). Alternative immunosuppressants may also be used, often in combination with corticosteroids (Colopy et al., 2010) or following corticosteroid treatment failure (Bennett, 1987; Itoh et al., 2010; and Kohn, 2007). Limited information exists on the efficacy of alternative immunosuppressants as standalone treatments. Corticosteroid treatment can cause significant adverse effects (Colopy et al., 2010; Miller, 1992; Perry, 2015; and Whitley & Day, 2011) and risks (Viviano, 2013). Since NSAIDs are contraindicated for use with corticosteroids (Boston et al., 2003; and Kohn, 2007), dogs undergoing treatment for immune-mediated polyarthritis have limited options for concurrent anti-inflammatory pain relief. Exploration of alternative immunosuppressive therapies is warranted.

Single case studies (Eom et al., 2015; and Wilson-Wamboldt, 2011) and case series with insufficient data (Clements et al., 2004; Colopy et al., 2010; Itoh et al., 2010; and Kohn et al., 2005) were excluded from this study due to low generalisability and power of conclusions. One clinically applicable primary research paper with reported promising results (Rhoades et al., 2016) was identified from the literature search, comparing cyclosporine to prednisone treatment in canine IMPA type I patients.

Rhoades et al. (2016) reported successful treatment for 70% (7/10) of dogs in both cyclosporine and prednisone groups. Of the three reported treatment failures in the cyclosporine group, two dogs were switched to prednisone (due to adverse effects or lack of improvement) and achieved resolution of symptoms. Consequently, at the end of the study period, nine dogs from both groups had been successfully treated with prednisone alone. Additionally, of the two dogs in the prednisone group switched to combination therapy, only the dog given prednisone and azathioprine achieved full symptom resolution, whilst the dog on prednisone and cyclosporine had persistent cytologic abnormalities. Although owner-reported and clinical signs of IMPA are informative (Colopy et al., 2010), dogs with IMPA may not show obvious discomfort or lameness (Bennett, 1987; and Jacques et al., 2002), therefore cytological results are thought to be the most reliable indicators of disease severity and improvement (Johnson & Mackin, 2012; Kohn, 2007; and Perry, 2015). Therefore, although 70% of both treatment groups achieved treatment success, prednisone appeared to be more successful than cyclosporine overall.

There were discrepancies in data collection that were not clearly explained in the text. Adverse effects data for cyclosporine dogs was reported for only five of the sample dogs by day 90. The loss of two of the remaining cyclosporine-treated dogs by day 90 means their contribution to adverse effects data was not evaluated by the end of the trial period. This emission is not explained in the text, but it is possible that two additional dogs were censored from adverse data collection due to the addition of concurrent medications for opportunistic infections. As the adverse effects data is the only table that includes sample size, it is unclear whether cytological and clinical data were collected from these dogs at day 90. Adverse effects data for the prednisone-treated dogs also introduced confusion, with adverse effects data being collected from nine dogs by the end of the study period, suggesting the two dogs switched from cyclosporine to prednisone treatment contributed to adverse effects data for the prednisone treatment group. This lack of clarity introduces uncertainty about reported conclusions.

Simple randomisation was used by Rhoades et al. (2016) by drawing cards from a hat. Selection bias is reduced through this process, but balanced distribution of population attributes or confounding variables cannot be achieved. With small samples, the risk of group differences being influenced by confounding variables is high. Rhoades et al. (2016) reported no statistical differences between groups in any of the variables measured, though it is unclear whether regression analyses were performed to ensure similarity. Additionally, the loss of dogs from both treatment groups over the trial period potentially created between-group differences not initially present. Nevertheless, since canine demographical variables have not been found to affect risk of immune-mediated arthritis (Clements et al., 2004; Colopy et al., 2010; Jacques et al., 2002; Johnson & Mackin, 2012; Kohn, 2007; and Stull et al., 2008) it may be that the study’s selection criteria sufficiently reduced the risk of confounding variables affecting results.

Blinding is important in controlled clinical trials to reduce the risk of conscious and unconscious bias. The follow-up regimes required for prednisone (gradual taper) versus cyclosporine (no taper, blood tests for trough concentration calculation) means Rhoades et al. (2016) were unable to blind clinicians or owners to treatment. The known and observable side effects of corticosteroids also impact the study’s ability to prevent awareness of treatment condition. The pathologists analysing fluid samples were blinded, reducing influence of bias in their cytologic results. However, few of the subjective owner-reported and clinician-reported variables correlated with objective pathologist-obtained variables across the duration of study, which suggests a problem with either the validity of the subjective ratings or the objective results.

Rhoades et al. (2016) did not control the use of concurrent medications. Other medications could have influenced adverse effect data throughout. In addition, cyclosporine-treated dogs were prescribed pain relief for the first 7 days of treatment, whilst prednisone-treated dogs were not. This regime choice was unexplained but may have been designed to balance pain relief across treatments. Unlike cyclosporine, prednisone does have anti-inflammatory properties. However, this early provision of pain relief may not have impacted the post-treatment data collection, which started 7 days after cessation of analgesics.

In conclusion, Rhoades et al. (2016) provided some support for the efficacy of cyclosporine, in comparison to prednisone, as a treatment of type I IMPA in some dogs. The study sample size and design concerns limit the strength of the findings. The reported treatment response of the dogs in the study population suggests that prednisone was somewhat more effective than cyclosporine, since some cyclosporine-treated dogs were later successfully treated with prednisone. More robust comparative studies are needed to support the efficacy of alternative immunosuppressive agents. This study does provide weak evidence that other immunosuppressants may provide effective treatment if corticosteroids are contraindicated, (eg. for unacceptable adverse effects) (Colopy et al., 2010; Itoh et al., 2010; Kohn, 2007; Mackin et al., 2016; Viviano, 2013; and Whitley & Day, 2011). Currently, there is not enough evidence from controlled clinical trials to warrant change to clinical practice.

## Methodology

**Search Strategy:** 

Databases searched and dates covered	CAB Abstracts on OVID Platform (1973–2021 Week 50) Web of Science – Core Collection (results from 1983–2022) Scopus (results from 1979–2022)
Search strategy	CAB Abstracts (keywords): [polyarth* and (immun* or inflammat* or immune-mediated) and (nonerosive or idiopathic or "type I" or IMPA) and (dog* or canid* or canine* or “Canis Familiaris”) and (treat* or corticosteroid* or immunosuppress* or immunomodulat* or azathioprine or prednis* or leflunomide or cyclosporine or levamisol or methotrexate or mycophenolate or cyclophosphamide or cytoxic or steroid* or medicat*)] Web of Science (topics): TS=[(polyarth* and (immun* or inflammat* or immune-mediated or nonerosive or idiopathic or "type I" or IMPA)) and (dog* or canid* or canine* or “Canis Familiaris”) and (treat* or corticosteroid* or immunosuppress* or immunomodulat* or azathioprine or prednis* or leflunomide or cyclosporine or levamisol or methotrexate or mycophenolate or cyclophosphamide or cytoxic or steroid* or medicat*)] Scopus (title, abstract, keywords): ([ TITLE-ABS-KEY ( polyarth* ) AND TITLE-ABS-KEY ( immun* OR inflammat* OR immune-mediated OR noncorrosive OR idiopathic OR "type I" OR impa ) AND TITLE-ABS-KEY ( treat* OR medicat* OR immunosuppress* OR immunomodulat* OR medicat* ) AND TITLE-ABS-KEY ( azathioprine OR leflunomide OR cyclosporine OR levamisole OR methotrexate OR mycophenolate OR cyclophosphamide OR cytoxic ) AND TITLE-ABS-KEY ( corticosteroid* OR prednis* OR steroid* ) AND TITLE-ABS-KEY ( dog* OR canid* OR canine* OR “Canis Familiaris”) ( DOCTYPE , "ar" )]
Dates searches performed	01 Sep 2022

**Exclusion / Inclusion Criteria:** 

Exclusion	Other types of IMPA (non-idiopathic), studies with no control or comparative group, non English-language, popular press, single case studies.
Inclusion	Any comparative systematic review / meta-analysis / randomised controlled clinical trial / cohort study that includes both corticosteroid (control) and alternative immunosuppressant (intervention) treatment.

**Search Outcome:** 

Database	Number of results	Excluded – Lack of relevance to clinical question	Excluded – Non-English language	Excluded – Not meeting criteria for controlled clinical trial	Excluded – Restricted access	Excluded – Duplicate paper	Total relevant papers
CAB Abstracts	40	34	0	4	0	1	1
Web of Science	75	67	0	6	0	1	1
Scopus	32	24	3	4	0	0	1
Total relevant papers when duplicates removed							1

## ORCID

Hannah Walker: https://orcid.org/0000-0003-0941-9847

## Conflict of Interest

The author declares no conflicts of interest.
